# Comparing Cognitive and Somatic Symptoms of Depression in Myocardial Infarction Patients and Depressed Patients in Primary and Mental Health Care

**DOI:** 10.1371/journal.pone.0053859

**Published:** 2013-01-14

**Authors:** Nynke A. Groenewold, Bennard Doornbos, Marij Zuidersma, Nicole Vogelzangs, Brenda W. J. H. Penninx, André Aleman, Peter de Jonge

**Affiliations:** 1 Interdisciplinary Center for Psychopathology and Emotion regulation, Department of Psychiatry, University Medical Center Groningen, Groningen, The Netherlands; 2 BCN Neuroimaging Center, Department of Neuroscience, University of Groningen, Groningen, The Netherlands; 3 Department of Psychiatry and EMGO Institute for Health and Care Research, VU University Medical Center, Amsterdam, The Netherlands; 4 Department of Psychiatry, Leiden University Medical Center, Leiden, The Netherlands; Harvard School of Public Health, United States of America

## Abstract

Depression in myocardial infarction patients is often a first episode with a late age of onset. Two studies that compared depressed myocardial infarction patients to psychiatric patients found similar levels of somatic symptoms, and one study reported lower levels of cognitive/affective symptoms in myocardial infarction patients. We hypothesized that myocardial infarction patients with first depression onset at a late age would experience fewer cognitive/affective symptoms than depressed patients without cardiovascular disease. Combined data from two large multicenter depression studies resulted in a sample of 734 depressed individuals (194 myocardial infarction, 214 primary care, and 326 mental health care patients). A structured clinical interview provided information about depression diagnosis. Summed cognitive/affective and somatic symptom levels were compared between groups using analysis of covariance, with and without adjusting for the effects of recurrence and age of onset. Depressed myocardial infarction and primary care patients reported significantly lower cognitive/affective symptom levels than mental health care patients (*F* (2,682) = 6.043, *p = *0.003). Additional analyses showed that the difference between myocardial infarction and mental health care patients disappeared after adjusting for age of onset but not recurrence of depression. These group differences were also supported by data-driven latent class analyses. There were no significant group differences in somatic symptom levels. Depression after myocardial infarction appears to have a different phenomenology than depression observed in mental health care. Future studies should investigate the etiological factors predictive of symptom dimensions in myocardial infarction and late-onset depression patients.

## Introduction

Research on myocardial infarction (MI) has demonstrated that dysfunction of the heart and dysfunction of emotion are closely related. Depression is a risk factor for both the onset and progression of cardiovascular disease [1]. Moreover, the 12-month prevalence of major depression is approximately two to four times higher in MI patients than in the general population [2]–[4]. A possible explanation for the high prevalence of depression is that somatic complaints as a consequence of the MI confound depression scores [5], [6]. Alternatively, symptoms of depression may be misattributed to the cardiac disease and go unnoticed [7]. Severe physical and psychological stress associated with MI might trigger the onset of depressive symptoms in individuals that have no pre-existing vulnerability for depression [8], [9]. This raises the question whether depression in MI patients is similar to depression as it is observed in psychiatric care. Several studies have therefore investigated the presentation and disease characteristics of depression in MI patients, or the phenomenology of post-MI depression.

In general depressed populations, studies support a distinction between somatic symptoms (e.g. fatigue, psychomotor abnormalities) and cognitive/affective symptoms of depression (e.g. feelings of guilt, depressed mood) [10], [11]. These symptom dimensions have also been reported in post-MI depression [6], [12], [13]. Two studies compared the symptom profiles of depressed MI patients and psychiatric patients. The first study reported comparable somatic symptom levels, but a lower number of cognitive/affective symptoms and depressive cognitions in a sample of 40 depressed MI patients compared to 40 depressed patients from psychiatric care [7]. Another study found comparable somatic symptom levels after adjusting for the number of cognitive/affective symptoms [14]. These results do not necessarily suggest an increase in somatic symptoms as the driving force of post-MI depression. Depression in MI patients might have a different phenomenology as depression in psychiatric care patients, but the difference is in cognitive/affective symptoms rather than in somatic symptoms.

Moreover, the developmental course of depressive symptoms may be different in MI patients than in psychiatric care patients. In the majority of cases, post-MI depression is reported to be a first episode (e.g. [15]). The age of onset in post-MI depression consequently is relatively high, considering the median age of onset of major depression in the general population is around 25 years [2]. This provides further support for a different phenomenology of depression in MI patients. In return, the symptom profile of depression may be associated with these characteristics. First, MI patients experiencing recurrent depression may have been previously treated for their depression in psychiatric care, and therefore their symptom profile might be more comparable to psychiatric care patients [8]. Second, a relationship between higher age of depression onset and fewer cognitive/affective symptoms has been previously established in depressed patients [16]. Therefore, characteristics of depression history may have influenced the results in previous studies on the symptom profile of depression in MI patients.

The primary aim of this study was to investigate whether MI patients have different levels of cognitive/affective and somatic symptoms than depressed patients without cardiovascular disease. Secondly, the effects of recurrence and age of depression onset on the symptom profile were examined. The hypothesis was that MI patients with a first episode of depression would report less cognitive/affective symptoms than depressed patients from primary and mental health care, and that this would be associated with a late onset of depression.

## Materials and Methods

### Study Design and Participants

To contrast the symptom profiles of depressed MI patients and depressed outpatients from primary care and mental health care, data from two large multicenter studies on depression conducted in the Netherlands were combined.

The Myocardial Infarction and Depression–Intervention Trial (MIND-IT) is a randomized clinical trial that was previously described in detail [17]. The goal of this study was to investigate the effects of psychiatric treatment on cardiac prognosis in MI patients with major depression. Recruitment took place between 1999 and 2002. In total, 2177 patients were screened with the Beck Depression Inventory (BDI; [18]) at 3, 6, 9 and 12 months after MI. At each time point, a BDI score ≥10 was followed by a structured diagnostic interview, the Composite Interview Diagnostic Instrument [19] (total *n = *799). MI patients with a diagnosis of major depressive disorder were included in the intervention trial and no longer took part in the screening procedure. The screening procedure continued three months later with the MI patients without a diagnosis of major depressive disorder.

For the current study, data from all time points were combined. All patients that experienced a major depressive episode in the month before the diagnostic interview were identified (*n = *211). Only patients with complete data on baseline descriptive characteristics were included in the final analyses (*n = *194). Of these patients, 76% experienced a first episode of depression (*n = *147).

The Netherlands Study of Depression and Anxiety (NESDA) is a longitudinal cohort study that is described in detail elsewhere [20]. NESDA investigated psychosocial and biological factors that influence the course of depressive and anxiety disorders. Recruitment took place between 2004 and 2007. In total, 2,981 participants were recruited from three different settings: the community (*n* = 564), primary care (*n* = 1,610) and specialized mental health care (*n* = 807). In primary care, patients were included through a screening procedure using the extended Kessler-10 [21]. Patients from mental health care were included when newly enrolled. All participants completed a structured diagnostic interview, the Composite Interview Diagnostic Instrument [19].

For the current study, only patients from primary care (PC) and specialized mental health care (MHC) were included, as the group of depressed people from the community was too small to consider separately (*n* = 33 participants meeting diagnostic criteria in the past month). Moreover, the community sample was selected to be an at-risk group and because of the different recruitment procedure, this group was not comparable with the other care groups. The mental health care group would be most comparable to previous studies on symptom profiles that included a psychiatric group. In addition, it was investigated whether the differences in cognitive/affective symptom levels would generalize to a primary care group. Similar to the MIND-IT sample, all patients that experienced a major depressive episode in the month before the interview were identified (primary care: *n* = 230; mental health care: *n* = 342). Finally, 7% of patients from PC and 5% of patients from MHC were excluded because of self-reported history of cardiac disease, i.e. myocardial infarction, coronary disease, angina pectoris, heart failure, cardiac arrhythmia, artery stenosis, or valvular disease. This selection was performed without verification of medication use, to be conservative in excluding all potential cardiac patients. This resulted in a sample of 214 depressed patients from PC and 326 patients from MHC.

### Ethics Statement

Both studies were conducted according to the principles expressed in the declaration of Helsinki. The study protocols from both MIND-IT and NESDA were approved by ethical review boards on human research of the collaborating institutions. All participants were provided with full written and oral information about the study procedure before written informed consent for study participation was obtained.

### Diagnostic Measures

Both studies used the Composite Interview Diagnostic Instrument (CIDI version 2.1; [19]) as diagnostic instrument. This interview was developed by the World Health Organization (WHO) in 1990. It is a structured clinical interview that contains questions directly corresponding to the symptoms of axis I psychiatric disorders listed in the Diagnostic and Statistical Manual of mental disorders (DSM-IV-TR; APA, 2004), for example: “In the past month, have you had two weeks or longer when nearly every day you felt sad, empty or depressed for most of the day?”, and “In the past month, have you had two weeks or longer when you lost interest in most things like work, hobbies and other things you usually enjoyed?”. The participant was asked to answer the questions with yes or no. Symptoms and the resulting disability were assessed to establish lifetime and current psychiatric diagnoses. The reliability of the CIDI is good and the validity is satisfactory for research purposes [19].

For this study, current depression was defined as meeting DSM-IV criteria for major depressive episode in the past month. Because of the special interest in cognitive/affective and somatic symptoms of depression, sum scores for each dimension were calculated from the relevant CIDI symptoms. The partitioning of depressive symptoms was based upon the factor analysis performed on the Patient Health Questionnaire 9 by De Jonge and colleagues [22], which includes the same 9 depression symptoms as the CIDI (PHQ-9; [23]). The cognitive/affective sum score included symptoms of depressed mood, anhedonia, feelings of guilt, concentration difficulties and thoughts of death (range: 1–5). The somatic sum score included symptoms of appetite/weight change, sleep abnormalities, psychomotor abnormalities and fatigue (range: 0–4). The standard questions from the CIDI provided additional data on the number of previous episodes of depression and age at onset. History of depression was determined retrospectively in both groups, as is common in post-MI depression studies [15], [24]. Demographic information was available from the interviews. Age and sex were selected as covariates. In MIND-IT, information about vascular risk factors was taken from the medical records during hospitalization. In NESDA it was assessed by means of self-report. Hypertension and diabetes were only regarded to be present when medication was necessary. In addition, history of cerebrovascular disease, current smoking and a high Body Mass Index (BMI; kg/m^2^) were considered to be vascular risk factors.

### Statistical Analyses

Statistical analyses were performed in SPSS for Windows, PASW Statistics 18 (SPSS Inc., Chicago, MA, USA). For the NESDA participants, all data on sex, age and CIDI symptoms were complete. There were 20 MI patients (13.6% of sample) with missing data on one or more CIDI depressive symptoms. For appetite or weight change, 5.4% of data was missing. For sleep abnormalities, 3.4% of data was missing. For fatigue and feelings of guilt, 2.7% of data was missing. For anhedonia, psychomotor abnormalities, concentration difficulties and thoughts of death only one observation was missing (<0.1%). A multiple imputation approach [25] was adopted to replace these missing values. The automated logistic regression approach in SPSS 18 was used to create an imputation model including the 9 DSM-IV symptoms of depression, sex and age. Missing values were replaced by imputed values estimated from the observed values on the predictor variables. Statistical analyses were performed on ten imputed datasets. The results from the individual datasets were combined according to Rubin’s rules [25] implemented in SPSS. All results were replicated in the original non-imputed dataset.

Differences between groups in descriptive characteristics, vascular risk factors and depression characteristics were analyzed using χ^2^ tests for categorical variables and one-way analysis of variance (ANOVA) for continuous variables. The association between vascular risk factors and depressive symptom levels was examined to rule out any confounding of subclinical vascular disease in the primary and mental health care groups. To test the primary hypothesis of the study, we first evaluated whether groups differed on a theory-driven distinction between cognitive/affective and somatic symptoms. For this purpose, group differences between the different patient samples were tested with analysis of covariance (ANCOVA), adjusted for age, sex, and levels of the other symptom dimension, respectively the somatic or cognitive/affective dimension. Because Rubin’s rules were not available for analysis of covariance, the median F-value from the imputed datasets was reported here. Significant results were followed up by Bonferroni adjusted pairwise comparisons to examine which groups were different from each other. Next, the effects of recurrence and age of onset on symptom levels were examined by adding these as covariates to the analyses. In addition, we examined heterogeneity in symptom profiles using a data-driven approach. For this purpose, latent class analysis was performed in MPLUS 5 [26]. The endorsement of all 9 DSM-IV symptoms (yes/no) by the participants from all three care groups were combined as input to the analysis. One to five latent class models were explored. The final model was selected based upon the parsimony indexes Bayesian information criteria (BIC) and Akaike information criteria (AIC), complemented by results from the Lo – Mendell – Rubin adjusted likelihood-ratio test (LRT). Next, differences between groups in the proportions of class assignment were compared by means of logistic regression analysis. Binary variables were created to code for class membership. Next, class membership was selected as outcome variable and care group as predictor with MI patients as a reference group, taking sex and age into account as covariates. Analyses were repeated for every class separately. The previously established effects of depression history on symptom profile from the theory-driven approach were additionally examined in the data-driven approach.

## Results

### Group Characteristics

A comparison of group characteristics showed substantial differences in demographics, vascular risk factors and depression characteristics (Table 1). As expected, MI patients were older and more often male than the other depressed patients. Depressed MI and PC patients presented with a lower total number of depressive symptoms than depressed patients from MHC, indicating a less severe type of depression. Depressed MI patients reported a higher age of depression onset and more often reported a first episode than the other depressed patients. Depressed MI patients with a first episode reported an average of 3.52 cognitive/affective symptoms compared to 3.70 for MI patients with a recurrent episode (*t* = −1.065, *df* = 185, *p = *0.29). Vascular risk factors were not associated with depressive symptom levels (all *p*>0.10).

**Table 1:** Group description – demographic characteristics, vascular risk factors and depression characteristics for depressed myocardial infarction, primary care and mental health care patients.

	MIND-IT	NESDA	NESDA		Posthoc
Variables of interest	All	PC	MHC	*P*-value	Tukey HSD
	N = 194	N = 214	N = 326		*p*<0.05
Female gender, %	25.3	69.6	66.0	<.001	
Age at testing, m (SD)	56.7 (11.1)	45.5 (12.2)	38.8 (11.0)	<.001	MI>PC>MHC
CVD, %	5.7	3.7	0.9	0.006	
Diabetes mellitus, %	13.0	4.2	4.0	<.001	
Hypertension, %	36.3	14.5	6.4	<.001	
Current smoker, %	56.0	44.9	48.5	0.032	
Previous smoker, %	23.8	32.2	23.3	-	
BMI, m (SD)	26.9 (4.1)	26.5 (5.2)	26.1 (5.8)	0.244	
CIDI symptoms, m (SD)	6.4 (1.2)	6.7 (1.3)	7.1 (1.3)	<.001	MI = PC<MHC
1. Sadness, %	91.8	81.8	83.7	0.010	
2. Anhedonia, %	77.7	86.4	94.2	<.001	
3. Appetite, %	45.7	63.6	64.4	<.001	
4. Sleep, %	84.0	87.4	86.2	0.622	
5. Psychomotor, %	75.1	66.8	71.5	0.178	
6. Fatigue, %	86.8	89.7	93.3	0.050	
7. Guilt feelings, %	52.6	57.5	66.6	0.005	
8. Concentration, %	83.4	95.8	98.5	<.001	
9. Thoughts death, %	51.0	43.0	50.6	0.159	
Age of onset, m (SD)	54.0 (11.6)	28.8 (12.8)	26.4 (10.9)	0.145	MI>PC = MHC
Recurrence, %	24.2	53.8	47.4	<.001	

Abbreviations: PC primary care, MHC mental health care, HSD honestly significant difference, CVD cerebro vascular disease, BMI body mass index, CIDI total number of depressive symptoms (range: 5–9) as established by composite interview diagnostic instrument, COG cognitive/affective, SOM somatic. Group differences were tested by means of ANOVA and χ^2^-test as appropriate.

### Analysis 1: Group Differences in Cognitive/Affective and Somatic Symptom Levels (Theory-driven Approach)

**Figure 1:**
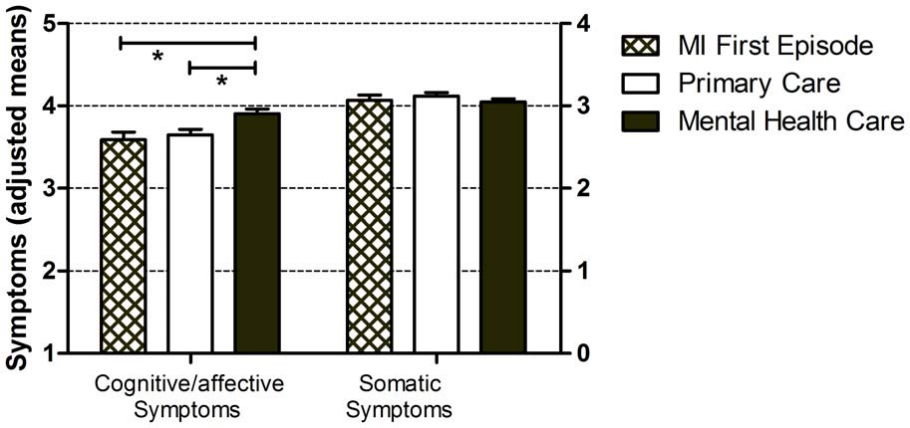
Group differences in cognitive/affective and somatic symptoms, comparing MI patients with first onset depression, depressed primary care and mental health care patients. * Means adjusted for age, sex and somatic symptom levels different at p<0.05, Bonferroni corrected.

Analysis of covariance (ANCOVA) adjusting for differences in age, sex and somatic symptom levels revealed a group difference in the number of cognitive/affective symptoms (*F* (2,681) = 5.821, *p = *0.003). Bonferroni corrected pairwise comparisons confirmed that MI patients experiencing a first episode of depression reported fewer cognitive/affective symptoms than MHC patients (3.590±0.093 and 3.906±0.057, *p* = 0.02) but not than PC patients (3.590±0.093 and 3.649±0.066, n.s.). Moreover, PC patients also reported fewer cognitive/affective symptoms than MHC patients (*p* = 0.01). There were no significant differences in somatic symptom levels (*F* (2,681) = 2.519, *p = *0.08). Similar results were obtained without adjusting for the other symptom dimension, with a group difference for cognitive/affective symptom levels (*F* (2,682) = 6.115, *p = *0.002) but not somatic symptom levels (*F* (2,682) = 2.644, *p = *0.07). The group differences in symptom levels are depicted in Figure 1, by means of adjusted means and standard errors. The results were highly comparable for the original and imputed datasets.

### Analysis 2: Effects of Depression History on Depressive Symptom Levels (Theory-driven Approach)

Subsequent sensitivity analyses were conducted to further examine the effects of recurrence and age of depression onset on cognitive/affective symptom levels. First, we repeated the ANCOVA on cognitive/affective symptoms including only the patients from PC (*n = *96) and MHC (*n = *170) experiencing a first episode of depression as control groups. The group differences were fully explained by age (*F_group_* (2,407) = 0.704, *p = *0.50 and *F_age_* (1,407) = 6.391, *p = *0.01). This is in contrast with the original analysis including patients with recurrent episodes of depression in the PC and MHC groups, where group was predictive of cognitive/affective symptom levels, but age was not (*F_age_* (1,681) = 2.107, *p = *0.15).

Next, we repeated the ANCOVA including depressed patients with first and recurrent episodes from the MI, PC and MHC groups. This analysis yielded a significant effect of group (*F_group_* (2,728) = 5.075, *p = *0.01), however not of age (*F_age_* (1,728) = 3.165, *p = *0.08). Indeed, when age of onset was included as a covariate, there was a significant effect of both group (*F_group_* (2,722) = 4.934, *p = *0.007) and age of onset (*F_ageons_* (1,722) = 13.529, *p*<0.001). Therefore, age of onset was a stronger predictor of cognitive/affective symptom levels than recurrence or age. The contrasts between the groups showed that the difference between depressed MI and MHC patients was no longer significant (3.754±0.088 and 3.865±0.058, n.s.). The PC group displayed the lowest levels of cognitive/affective symptoms (3.593±0.068), which was significantly lower than the MHC group (*p* = 0.005).

### Analysis 3: Heterogeneity in Symptom Profiles Established by Latent Class Analysis (Data-driven Approach)

**Figure 2:**
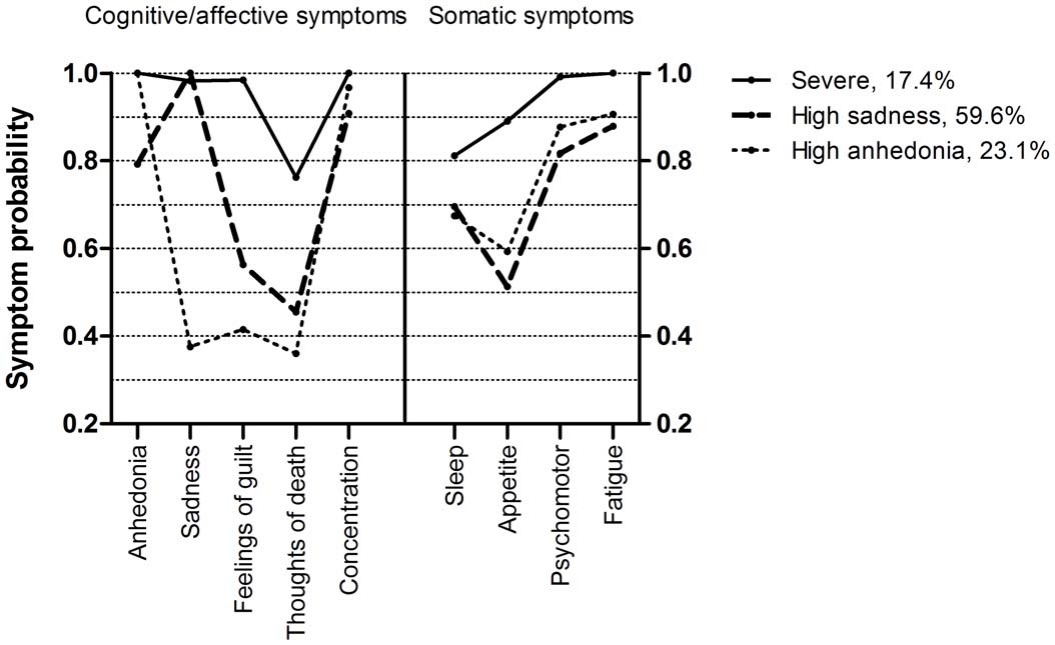
The three different symptom profiles of depression established by latent class analysis.

A three-class solution provided the best model fit (AIC = 6382, sample-size adjusted BIC = 6424, compared to AIC = 6414, sample-size adjusted BIC = 6441 for a two-class solution). The LRT confirmed that a four-class solution did not have additional explanatory value to a three-class solution (*p = *0.10 compared to a previous *p = *0.03). The first class was a class with severe depression, characterized by a high probability of endorsement of each DSM-IV symptom. The other two classes were of moderate severity, having a lower probability of reporting three cognitive/affective symptoms (i.e. one of the core symptoms, feelings of guilt and thoughts of death) and one somatic symptom (appetite changes). Furthermore, the low cognitive classes could be distinguished by the core symptoms of depression. Class 2 reported more sadness than class 3, whereas class 3 reported more anhedonia than class 2. The symptom profiles of the three classes are depicted in Figure 2.

**Figure 3:**
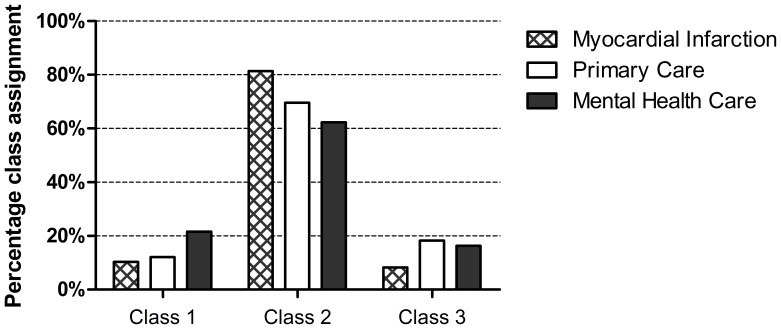
Percentage of class assignment for depressed myocardial infarction, primary care and mental health care patients. N.B. Class 1: Severe, Class 2: Low cognitive – high sadness, Class 3: Low cognitive – high anhedonia.

Next, the individual participants were assigned a most likely class membership. The frequencies of class assignment for the different depression groups are depicted in Figure 3. Logistic regression analyses were performed to compare class assignment in depressed MI patients to depressed patients from primary and mental health care, adjusting for age and sex. MI patients had lower odds of being classified as having a profile of severe depression than MHC patients (OR = 0.519, *p* = 0.046). The severe class was equally represented in the MI and PC groups. The odds of low cognitive – high sadness class assignment were higher in the MI group than in the PC (OR = 1.649, *p* = 0.056) and MHC group (OR = 2.201, *p* = 0.002), although the difference with the PC group was only marginally significant. The odds of low cognitive – high anhedonia class assignment tended to be lower in the MI group than in the PC (OR = 0.426, *p* = 0.013) and MHC group (OR = 0.509, *p* = 0.054). The results are summarized in Table 2.

**Table 2:** Odds ratio of symptom profile class membership in depressed MI patients compared to patients from primary and mental health care, controlled for age and sex.

Symptom profile	Comparison group	Odds ratio	95% Confidence Interval	P-value
Severe depression	Primary Care	1.002	0.508–1.976	0.996
	Mental Health Care	0.519	0.273–0.988	0.046
Low cognitive- high sadness	Primary Care	1.649	0.988–2.751	0.056
	Mental Health Care	2.201	1.324–3.659	0.002
Low cognitive-high anhedonia	Primary Care	0.426	0.217–0.837	0.013
	Mental Health Care	0.509	0.256–1.013	0.054

Note: The odds ratios represent group differences in the odds of being classified as having the specific symptom profile. For each comparison, the myocardial infarction (MI) patients group is the reference group.

Because age of onset was the best predictor in the theory-driven analyses, age of onset was included as an additional predictor in the logistic regression models. A higher age of onset showed a substantial, however only marginally significant association with the low cognitive – high anhedonia class (OR = 1.020, *p* = 0.057). Age of onset was not predictive for the severe or low cognitive – high sadness classes (*p*>0.2). For the severe class, the difference between the MI and MHC groups was slightly attenuated (OR = 0.547, *p* = 0.086) after including age of onset as a predictor. For the low cognitive – high sadness and low cognitive – high anhedonia classes, the MI group was now significantly different from the PC and MHC groups (all *p*<0.05).

## Discussion

This is the first study to investigate differences in symptom profile between depressed MI patients and depressed patients from primary and mental health care, and to examine the effects of characteristics of depression history. Overall, MI and PC patients reported fewer depressive symptoms than MHC patients. The hypothesis that MI patients would show fewer cognitive/affective symptoms than patients from MHC was confirmed. In addition, patients from PC also reported fewer cognitive/affective symptoms than patients from MHC. A later age of depression onset was related to lower cognitive/affective symptom levels. The differences between the MI and MHC groups were explained by differences in age of onset but not by recurrence of depression. The results from the theory-driven analyses on symptom dimensions were supported by the data-driven latent class analyses. MI patients more often demonstrated a low cognitive – high sadness profile than PC and MHC patients. This difference could not be explained by age of onset.

This is the first study to compare symptom profiles of depressed MI patients and depressed patients from PC and MHC with a large sample size (687 depressed individuals), providing adequate statistical power. In addition, the study used a structured clinical interview, which is a reliable measure of clinically relevant symptoms. More importantly, the analyses on symptom dimensions were hypothesis-driven, following a theoretical framework [8]. These analyses were complemented by a data-driven approach. Another strength of this study is the nature of the samples. MI is a cardiac condition with an unambiguous onset time, offering the opportunity to look at depressive symptoms experienced after the cardiac event. Two control groups of depressed patients were included in the analyses to investigate the generalizability of the differences in symptom profile.

There are also several limitations to this study. The most important limitation is its cross-sectional design. As a consequence, this study does not allow for any conclusions on the etiology of depressive symptoms. In addition, it was not possible to look at the development of symptom profiles over time. As history of depression was assessed retrospectively in both groups, recall bias might have influenced the results; however as the same method was used for all participants it would be present in all groups. The analyses were adjusted for sex and age, and sensitivity analyses were conducted to look at the effects of history of depression. Nevertheless, there may be other confounding factors that were not included in the analyses. Therefore it is important to examine the generalizability of the findings to patient samples with other cardiovascular problems or late-onset depression before definite conclusions can be drawn. The most evident confounding factor would be severity of depression. Unfortunately the two studies did not use the same instrument to measure depression severity (MIND-IT used the Beck Depression Inventory [18] and NESDA used the Inventory of Depressive Symptoms [27]). An alternative would be to adjust for the total number of symptoms. However, it is noteworthy that the number of cognitive/affective symptoms is part of, and therefore dependent upon, the total number of depressive symptoms. Accordingly, including severity as a covariate when looking at cognitive/affective symptom levels would lead to an unstable regression model and would by definition lead to overcorrection. As an alternative, we decided to adjust for the contrasting symptom dimension as a more independent measure, as was done previously [14].

The results of this study are complementary to previous findings. A lower prevalence of cognitive/affective symptoms with equivalent somatic symptoms in post-MI depression compared to depressed patients in mental health care is a direct replication of the findings of Martens and colleagues [7], but this time in a much larger sample. Similarly, equivalent somatic symptom levels have been reported comparing MI patients and a heterogeneous sample of psychiatric patients, matched on cognitive/affective symptom levels [14]. The current study confirms that somatic symptoms are not elevated in MI patients compared to other depressed patients. Moreover, the results from the latent class analysis suggest that cognitive and somatic symptom levels might be lower in MI and PC patients, possibly reflecting a general effect of severity. However, somatic complaints may still influence the somatic symptom levels in depressed MI patients. Whether the etiology behind the somatic symptoms is the same in depressed MI patients as in psychiatric patients remains to be determined.

Surprisingly, PC patients reported fewer cognitive/affective symptoms than patients from MHC. Therefore, the extra control group of depressed PC patients provided crucial information that was lacking in the previous studies. Not the MI group but the MHC group appears to be different from the others. There are several potential explanations for this finding. For instance, patients seeking treatment for their depression might be characterized by relatively high levels of cognitive/affective symptoms. Alternatively, MHC patients might have increased cognitive vulnerability for depression. It is important to note that the prevalence of depression in primary care may be equally high as the prevalence in MI patients [21]. Therefore, it might be interesting to examine the etiological factors predicting symptom dimensions in these patients as well. For instance, somatic complaints could contribute to the development of somatic symptoms in PC and MI patients. New studies on the symptom profiles of depression should take these findings into account and carefully consider which control groups need to be included.

The results regarding lower cognitive/affective symptoms in MI and PC patients were confirmed and complemented by data-driven symptom profile classification. The latent class analysis demonstrated that the most severe class was most prevalent in the MHC group. In addition, MI patients more often displayed the high sadness – low cognitive symptom profile and PC patients more often displayed the high anhedonia – low cognitive symptom profile. This remarkable shift in affective symptoms was also clearly present in the frequency of symptom endorsement (Table 1), and to our knowledge has not been reported before. Sadness appears to be the most important cognitive/affective symptom in MI patients. More sadness in MI patients might reflect a reactive emotional response to a major life event and deserves further investigation. Feelings of guilt, thoughts of death, and appetite changes were less prevalent in MI and PC patients. Factor analyses of depressive symptoms in MI patients have reported a separate factor of appetite changes [12], so it may not be very well classified within the cognitive or somatic symptom dimension. Less feelings of guilt were previously reported in a study in heart failure patients [28] but also in late-onset depression in the general population [29], [30].

**Figure 4:**
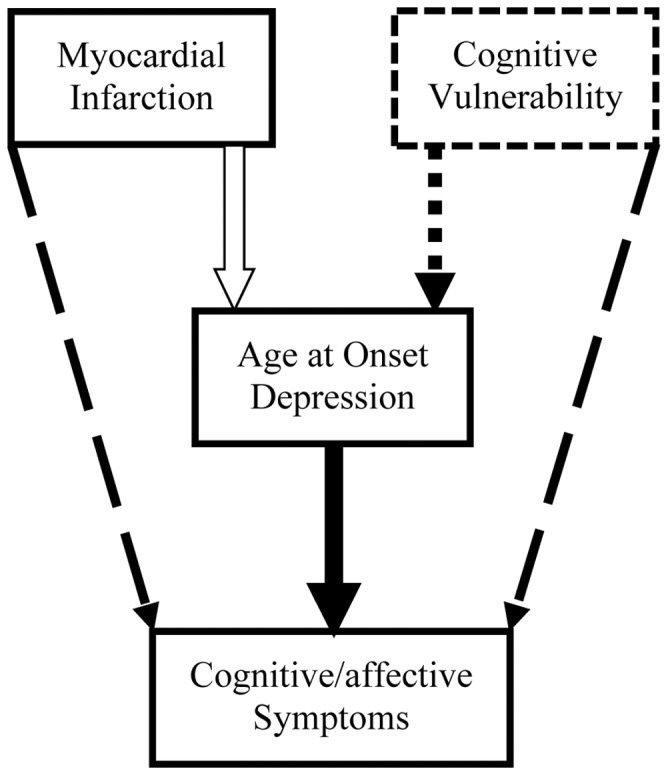
The hypothesized associations between age of depression onset, myocardial infarction and cognitive/affective symptom levels in depressed patients. N.B. White arrows denote a positive association and black arrows denote a negative association. Cognitive vulnerability is included as a potentially influential but in our study unmeasured factor.

As expected, MI patients reported a higher age of onset and less recurrence than PC and MHC patients. A lower age of onset rather than recurrence of depression was associated with higher cognitive/affective symptom levels. Adjusting for age of onset differences eliminated the difference between the MI and MHC groups. Previously, it has been proposed that low levels of cognitive/affective symptoms are associated with a relatively low pre-existing cognitive vulnerability for depression in patients with cardiovascular disease [8], [31]. One possible explanation for our findings is that pre-existing cognitive vulnerability might be less pronounced in depressed MI patients with a high age of onset, since these patients did not develop depression after stressors encountered prior to MI. Cognitive vulnerability is a well- established risk factor for early-onset depression [32], [33]. Interestingly, it has been found that late-onset depression is associated with lower levels of neuroticism and higher levels of stress than early-onset depression [34], [35]. Hence, patients who develop a first episode in late life may have a relatively low cognitive vulnerability for depression, irrespective of cardiovascular disease. The hypothesized associations between MI, age of onset and cognitive/affective symptoms are depicted in Figure 4.

Our study established similar levels of somatic symptoms between MI, PC and MHC patients and lower levels of cognitive/affective symptoms in MI and PC compared to MHC patients. Thereby, the MI and PC groups showed differences in affective symptom endorsement. We confirmed that post-MI depression has a different phenomenology than depression as it is observed in mental health care. Future research should investigate whether the etiological factors predicting depression are different as well. A possible explanation for the findings is that MI is such an influential stressor that even people with a low predisposing cognitive vulnerability become depressed. These findings may be the impetus for further research on the role of cognitive vulnerability and stress in late-onset depression. Furthermore, additional neurobiological processes might be influential (e.g. [36], [37]). Future studies should consider biological markers associated with somatic and cognitive/affective symptoms of depression, such as the physiological stress response, inflammation and brain abnormalities. For now, the idea of low cognitive vulnerability sheds a positive light on a complex disorder and may inspire research on protective factors as well as risk factors for depression.
